# Dataset on statistical analysis of editorial board composition of Hindawi journals indexed in Emerging sources citation index

**DOI:** 10.1016/j.dib.2018.02.044

**Published:** 2018-02-20

**Authors:** Hilary I. Okagbue, Aderemi A. Atayero, Muminu O. Adamu, Abiodun A. Opanuga, Pelumi E. Oguntunde, Sheila A. Bishop

**Affiliations:** a Department of Mathematics, Covenant University, Canaanland, Ota, Nigeria; b Department of Electrical and Information Engineering, Covenant University, Canaanland, Ota, Nigeria; c Department of Mathematics, University of Lagos, Akoka, Lagos, Nigeria

**Keywords:** Hindawi, Bibliometrics, Data analysis, ESCI, Random, Smart campus, Web of science, Ranking analytics, Statistics

## Abstract

This data article contains the statistical analysis of the total, percentage and distribution of editorial board composition of 111 Hindawi journals indexed in Emerging Sources Citation Index (ESCI) across the continents. The reliability of the data was shown using correlation, goodness-of-fit test, analysis of variance and statistical variability tests.

**Specifications table**

**Table:** 

Subject area	Decision Sciences
More specific subject area	Bibliometrics, Statistical data analysis
Type of data	Table, Figure and MS Excel
How data was acquired	The data was obtained from freely open access hindawi journals
Data format	Raw, partially analyzed
Experimental factors	Patterns of composition of editorial members of journals indexed in ESCI.
Experimental features	Only the Journals indexed in ESCI were considered
Data source location	Hindawi Publisher
Data accessibility	All the data are in this data article

**Value of the data**

• The data could be helpful in the determination of the impact of journal indexing on scientific publications.
• The analysis can be extended to other publishers.
• The dataset can be helpful in bibliometric analysis.
• The dataset can be helpful as a ranking analytics for journals and management of smart campuses.
• The dataset can be helpful in monitoring the impact of editorial composition in the acceptance and rejection of manuscripts submitted to different *Hindawi* journals.
• The dataset can provide insight to the following: stereotyping in academic publications, duration differences in acceptance or rejection of manuscript, bias in publication. See [1] for the case of management academic area.
• The dataset can spur academic discourse on the effect of geographical distribution of editorial board membership on perceived research output using the journals indexed in ESCI as case study. This can be achieved when citation analysis is incorporated. See the conclusions of [2].
• Several statistical models and methods can be applied to the dataset for further analysis.

## 1 Data

**Fig. 1:**
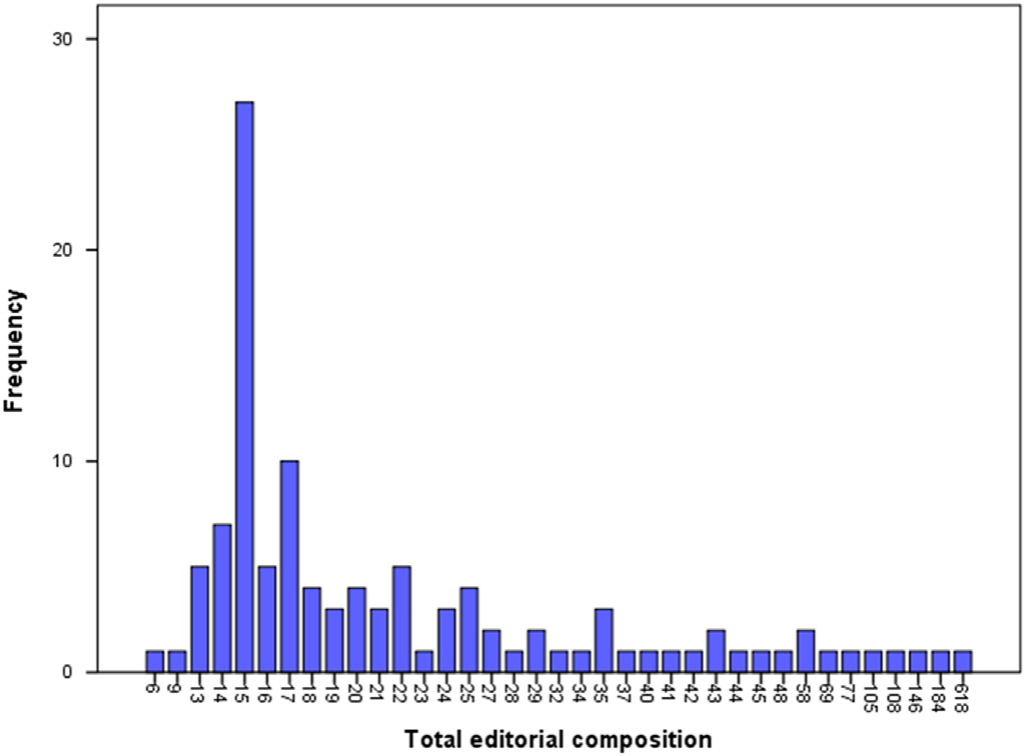
Total editorial composition of Hindawi journals indexed in ESCI.
c. The editorial board membership grouped into six continents. These are presented in bar charts. See Fig. 2**a, b, c, d** and **e**.

The dataset contained in this article are listed as follows:

a. The dataset of editorial composition of 111 Hindawi journals indexed in ESCI. This can be assessed as Supplementary data 1.
b. The frequency of editorial board composition of the 111 Hindawi journals and their summary statistics. This is presented in Fig. 1.

**Fig. 2:**
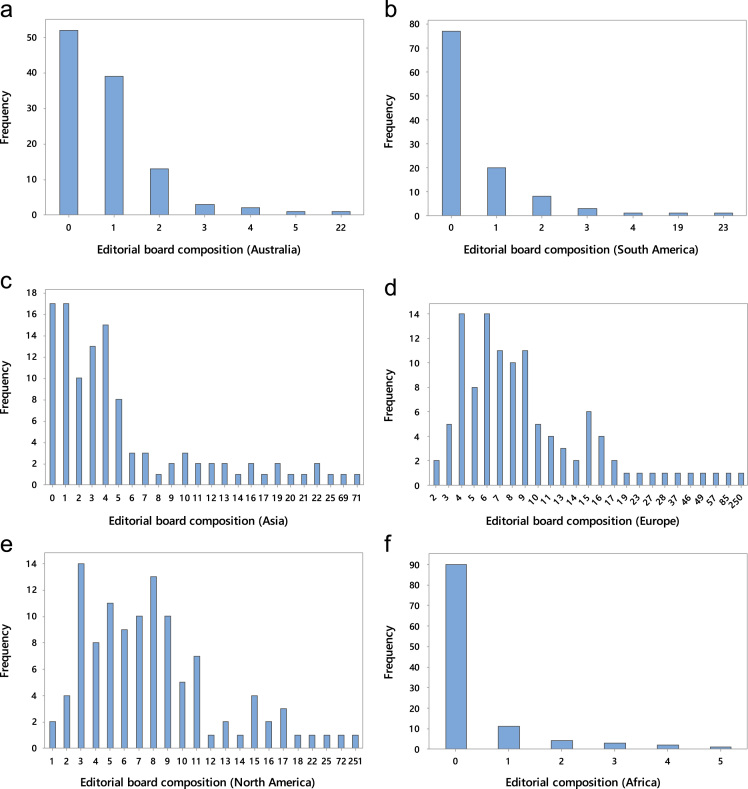
**a:** Editorial board composition from Australia. **b:** Editorial board composition from South America. **c:** Editorial board composition from Asia. **d:** Editorial board composition from Europe. **e:** Editorial board composition from North America. **f:** Editorial board composition from Africa.
d. The detailed statistical analysis such as correlation analysis, test of normality and analysis of variance
e. The detailed dataset showing the Poisson distribution goodness-of-fit test of the data classified into six continents namely North America (NAM), Europe (EURO), Asia (ASIA), South America (SAM), Australia (AUST) and Africa (AFR).

### 1.1 Detailed data description

Hindawi Publishing Corporation is one of the leading academic publishers of medical, technical, social and scientific peer-reviewed literature. Currently, they publish 302 journals that cut across different academic domains. The publisher operates on fully open access model under Creative Commons Attribution License (CC-BY). The editorial policies of the journals stipulate that they operate without editor-in-chief but rather through editorial boards. Manuscripts submitted to the journals are first processed at the editorial office and sent to an assigned editor chosen from the pool of editorial board members of the journal. The assigned editor is then saddled with the responsibility of sourcing for qualified reviewers for the manuscript. The decision to accept or reject solely rests on the shoulders of the editors. The business model used by the publisher is that indexing determines the article processing charges.

Currently, Hindawi publishing Corporation publishes 111 journals indexed in emerging sources citation index (ESCI). ESCI is part of web of science owned and maintained by of Clarivate Analytics (formerly Thomson Reuters). ESCI has been in existence since 2015 and it includes peer reviewed academic journals.

## 2 Experimental design, materials and methods

The experimental design used in this paper is the application of statistical methods targeted at revealing the hidden patterns of the datasets. Text mining was used to extract the dataset from the publisher's website. Similar analysis on statistical methods and the applications in bibliometrics can be found in Ref. [3], [4], [5], [6], [7], [8], [9], [10], [11], [12], [13], [14], [15], [16]. In addition, those works have helped in deeper understanding of pattern of editorial composition, citation analysis, rejection and acceptance rates and others.

### 2.1 Distribution of Editorial board membership (composition) across the six continents

The editorial board membership of the publisher is classified into six continents. The summary statistics is as shown in Table 1.

**Table 1:** Summary statistics of the distribution of editorial board membership of ESCI Hindawi journals across the continents.

		NAM	EURO	ASIA	SAM	AUST	AFR
N	Valid	111	111	111	111	111	111
	Missing	0	0	0	0	0	0
Mean		10.51	12.84	6.25	0.82	0.98	0.37
Std. Error of Mean		2.301	2.409	0.986	0.273	0.213	0.089
Median		7.00	8.00	3.00	0.00	1.00	0.00
Mode		3	4^a^	0^a^	0	0	0
Std. Deviation		24.244	25.380	10.392	2.877	2.240	0.933
Variance		587.779	644.137	107.990	8.276	5.018	0.871
Skewness		9.181	7.859	4.453	6.592	7.737	3.018
Std. Error of Skewness		0.229	0.229	0.229	0.229	0.229	0.229
Kurtosis		90.265	70.917	24.534	46.181	71.531	9.340
Std. Error of Kurtosis		0.455	0.455	0.455	0.455	0.455	0.455
Range		250	248	71	23	22	5
Minimum		1	2	0	0	0	0
Maximum		251	250	71	23	22	5
Sum		1167	1425	694	91	109	41
Percentiles	25	4.00	5.00	1.00	0.00	0.00	0.00
	50	7.00	8.00	3.00	0.00	1.00	0.00
	75	10.00	11.00	7.00	1.00	1.00	0.00
	90	15.00	17.00	16.00	2.00	2.00	1.00

**Remarks:** Most ESCI Hindawi journals does not have editors from Africa, Australia and South America.

a Multiple modes exist. The smallest value is shown.

### 2.2 Percentage editorial board membership composition

Percentage editorial board membership composition of Hindawi journals indexed in ESCI was obtained to show the actual percentage composition across the continents. This is shown in Table 2.

**Table 2:** Percentage and total number of editors across the continents.

Continent	Total	Percentage
North America	1167	33
Europe	1425	40.4
Asia	694	19.7
South America	91	2.6
Australia (oceanic)	109	3.1
Africa	41	1.2
Total	3527	100

### 2.3 Correlation

The correlation results using Spearman, Pearson and Kendall correlation coefficient are obtained in form of matrices shown in Table 3a, Table 3b, Table 3c.

**Table 3a:** A correlation matrix of the editorial board composition (Pearson correlation coefficient).

Variables	NAM	EURO	ASIA	SAM	AUST	AFR
NAM	1					
EURO	0.950754	1				
ASIA	0.655586	0.765639	1			
SAM	0.736201	0.775160	0.527298	1		
AUST	0.893556	0.870145	0.577012	0.714708	1	
AFR	0.308473	0.419262	0.495423	0.475230	0.285796	1

**Table 3b:** A correlation matrix of the editorial board composition (Spearman correlation coefficient).

Variables	NAM	EURO	ASIA	SAM	AUST	AFR
NAM	1					
EURO	0.076240	1				
ASIA	0.134405	0.540343	1			
SAM	0.101428	0.257115	0.294628	1		
AUST	0.242937	0.122644	0.095750	0.186542	1	
AFR	0.173750	0.254738	0.278440	0.316884	0.178483	1

**Table 3c:** A correlation matrix of the editorial board composition (Kendall correlation coefficient).

Variables	NAM	EURO	ASIA	SAM	AUST	AFR
NAM	1					
EURO	0.047338	1				
ASIA	0.090226	0.411477	1			
SAM	0.082719	0.213338	0.241630	1		
AUST	0.198969	0.096349	0.071850	0.169551	1	
AFR	0.142385	0.211057	0.231271	0.293869	0.167058	1

The distances between the correlations are computed using the following;

$$A_{1}=\left|Pearson-Spearman\right|$$

$$A_{2}=\left|Kendall-Pearson\right|$$

$$A_{3}=\left|Spearman-Kendall\right|$$

The application of the transformations and their percentages using Table 3a, Table 3b, Table 3c are presented in Table 4. Correlation analysis often reveals some interesting hidden pattern in data. See [17], [18], [19], [20], [21] for details.

**Table 4:** Absolute difference between the correlations coefficients and their percentages.

Variables	$A_{1}$	$A_{2}$	$A_{3}$	$\%A_{1}$	$\%A_{2}$	$\%A_{3}$
(NAM, EURO)	0.874514	0.903416	0.028902	87.4514	90.3416	2.8902
(NAM, ASIA)	0.521181	0.56536	0.044179	52.1181	56.536	4.4179
(NAM, SAM)	0.634773	0.653482	0.018709	63.4773	65.3482	1.8709
(NAM, AUST)	0.650619	0.694587	0.043968	65.0619	69.4587	4.3968
(NAM, AFR)	0.134723	0.166088	0.031365	13.4723	16.6088	3.1365
(EURO, ASIA)	0.225296	0.354162	0.128866	22.5296	35.4162	12.8866
(EURO, SAM)	0.518045	0.561822	0.043777	51.8045	56.1822	4.3777
(EURO, AUST)	0.747501	0.773796	0.026295	74.7501	77.3796	2.6295
(EURO, AFR)	0.164524	0.208205	0.043681	16.4524	20.8205	4.3681
(ASIA, SAM)	0.23267	0.285668	0.052998	23.267	28.5668	5.2998
(ASIA, AUST)	0.481262	0.505162	0.0239	48.1262	50.5162	2.39
(ASIA, AFR)	0.216983	0.264152	0.047169	21.6983	26.4152	4.7169
(SAM, AUST)	0.528166	0.545157	0.016991	52.8166	54.5157	1.6991
(SAM, AFR)	0.158346	0.181361	0.023015	15.8346	18.1361	2.3015
(AUST, AFR)	0.107313	0.118738	0.011425	10.7313	11.8738	1.1425

The result of the partial correlation is presented in Table 5.

**Table 5:** Partial correlation coefficients r.

Variables	$r_{1}$	$r_{2}$	$r_{3}$
(NAM, EURO, ASIA)	0.92396	−0.36286	0.60816
(NAM, EURO, SAM)	0.88896	−0.00400	0.35857
(NAM, EURO, AUST)	0.78299	0.43382	0.14799
(NAM, EURO, AFR)	0.95117	−0.32034	0.42729
(EURO, ASIA, SAM)	0.66486	0.67958	−0.16288
(EURO, ASIA, AUST)	0.65482	0.81534	−0.28140
(EURO, ASIA, AFR)	0.70747	0.07149	0.29866
(ASIA, SAM, AUST)	0.20115	0.33679	0.59146
(ASIA, AUST, AFR)	0.52308	0.42229	−0.00001
(SAM, AUST, AFR)	0.68657	0.40428	−0.08751

### 2.4 Goodness-of-fit test

**Fig. 3:**
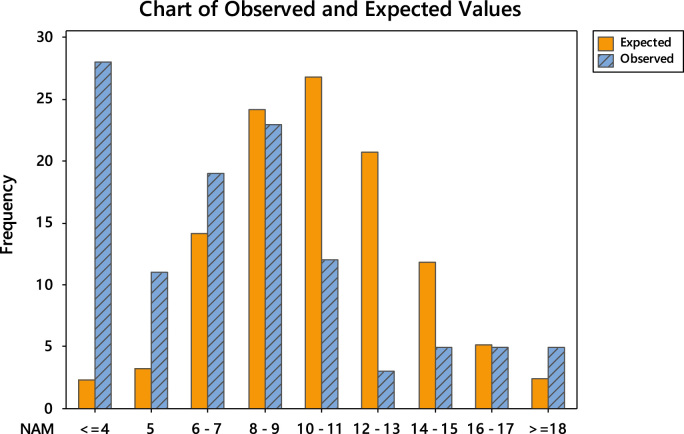
Chart of observed and expected values (North America).

**Fig. 4:**
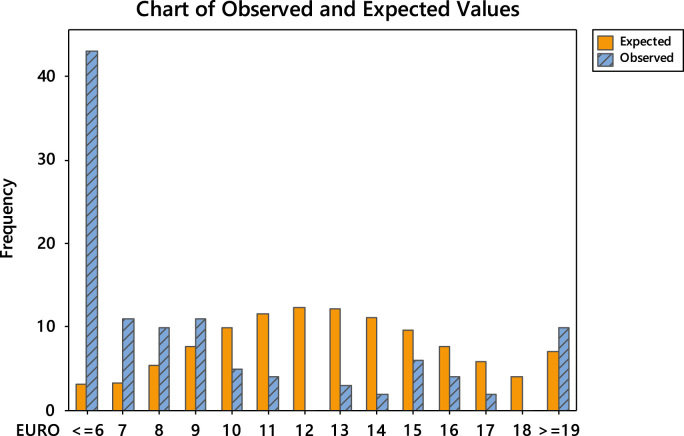
Chart of observed and expected values (Europe).

**Fig. 5:**
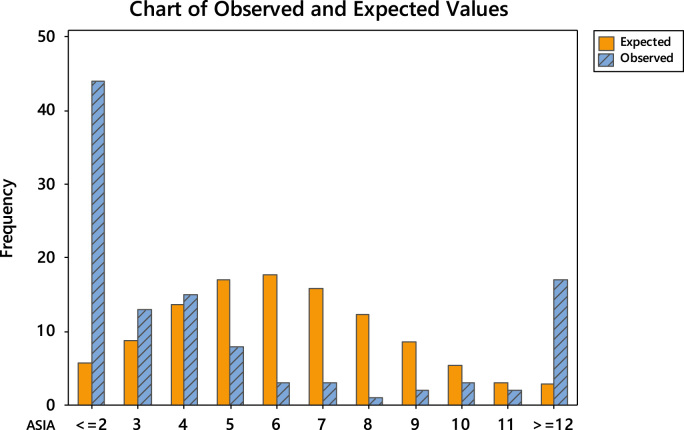
Chart of observed and expected values (Asia).

**Fig. 6:**
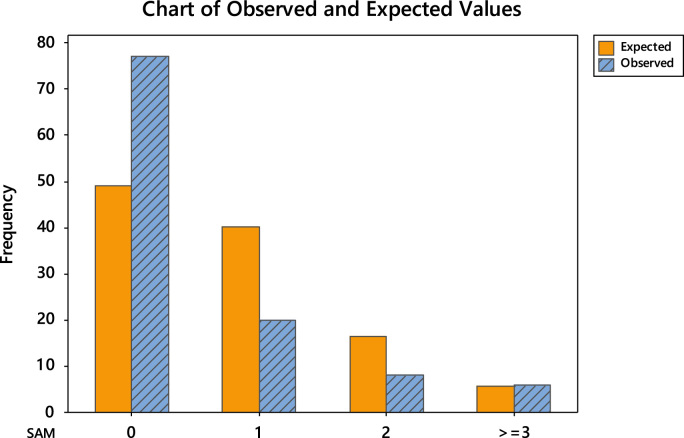
Chart of observed and expected values (South America).

**Fig. 7:**
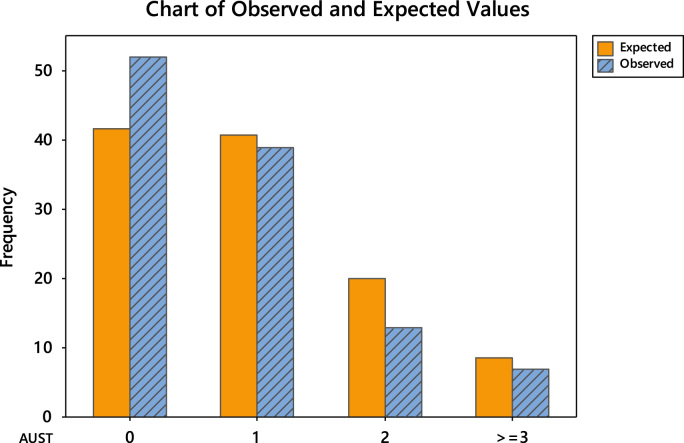
Chart of observed and expected values (Australia).

**Fig. 8:**
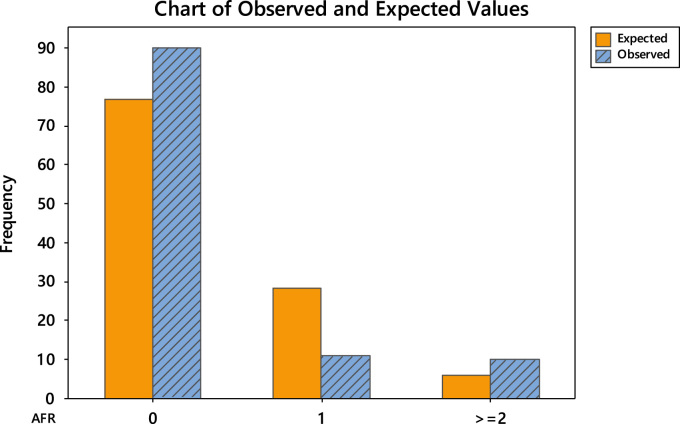
Chart of observed and expected values (Africa).

The uneven editorial board membership composition across the continents necessitated the conduct of goodness-of-fit test using Poisson distribution. The goodness-of-fit results are divided into two. Firstly, the detailed tests are shown in Table 6, Table 7, Table 8, Table 9, Table 10, Table 11 and the chart of the observed and expected values are shown in Fig. 3, Fig. 4, Fig. 5, Fig. 6, Fig. 7, Fig. 8.

**Table 6:** Goodness-of-fit test for Poisson distribution (North America).

**Table 7:** Goodness-of-fit test for Poisson distribution (Europe).

**Table 8:** Goodness-of-fit test for Poisson distribution (Asia).

**Table 9:** Goodness-of-fit test for Poisson distribution (South America).

**Table 10:** Goodness-of-fit test for Poisson distribution (Australia).

**Table 11:** Goodness-of-fit test for Poisson distribution (Africa).

### 2.5 Analysis of variance

**Fig. 9:**
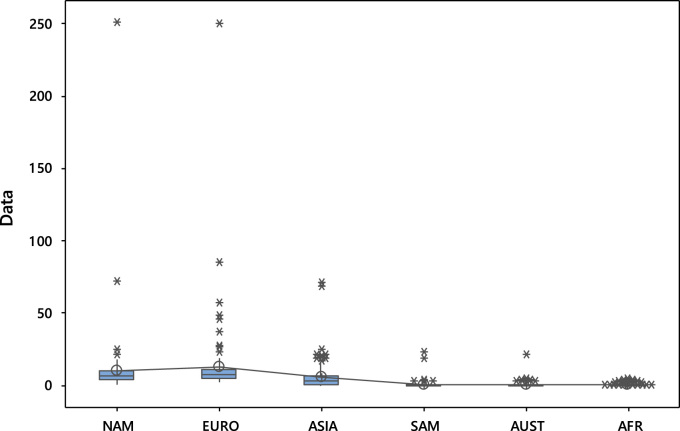
Box plot of editorial board composition across the continents.

**Fig. 10:**
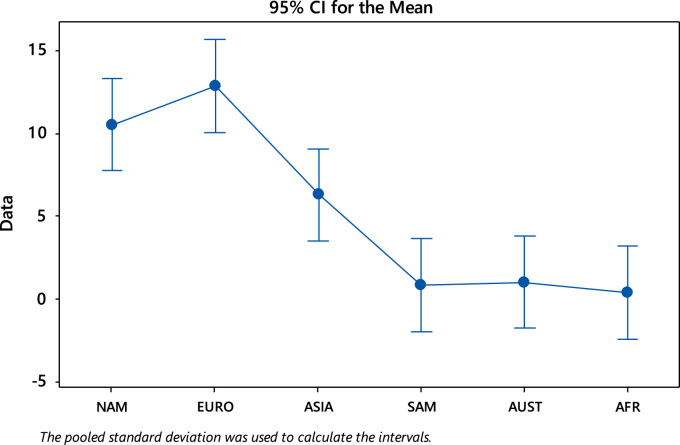
Interval plot of editorial board composition across the continents.

The data is subjected to analysis of variance (ANOVA) and the result is shown in Table 12. Furthermore the boxplot and interval plot of the data are displayed in Fig. 9, Fig. 10 respectively.

**Table 12:** Analysis of variance of the editorial board composition across the continents of ESCI indexed Hindawi journals.

### 2.6 Statistical variability analysis

Different variability measures are conducted for editorial board composition of ESCI indexed Hindawi journals across the continents. These are summarized in Table 13, Table 14, Table 15, Table 16, Table 17, Table 18.

**Table 13:** Variability analysis of the North America data.

Absolute range	250
Relative range (unbiased)	10.3118
Variance (unbiased)	587.779
Standard Deviation (unbiased)	24.2442
Coefficient of Variation (unbiased)	2.306
Squared Differences between all Pairs of Observations	1175.56
Mean Absolute Differences between all Pairs of Observations	10.2165
Gini Mean Difference	10.2165
Leik Measure of Dispersion	0.559367
Index of Diversity	0.943516
Index of Qualitative Variation	0.952093
Coefficient of Dispersion	1.0223
Observations	111

**Table 14:** Variability analysis of the Europe data.

Absolute range	248
Relative range (unbiased)	9.77153
Variance (unbiased)	644.137
Standard Deviation (unbiased)	25.3799
Coefficient of Variation (unbiased)	1.97696
Squared Differences between all Pairs of Observations	1288.27
Mean Absolute Differences between all Pairs of Observations	12.9369
Gini Mean Difference	12.9369
Leik Measure of Dispersion	0.56356
Index of Diversity	0.956098
Index of Qualitative Variation	0.96479
Coefficient of Dispersion	1.19229

**Table 15:** Variability analysis of the Asia data.

Absolute range	71
Relative range (unbiased)	6.83228
Variance (unbiased)	107.99
Standard Deviation (unbiased)	10.3918
Coefficient of Variation (unbiased)	1.6621
Squared Differences between all Pairs of Observations	215.981
Mean Absolute Differences between all Pairs of Observations	7.86306
Gini Mean Difference	7.86306
Leik Measure of Dispersion	0.510375
Index of Diversity	0.966327
Index of Qualitative Variation	0.975112
Coefficient of Dispersion	1.95157

**Table 16:** Variability analysis of the South America data.

Absolute range	23
Relative range (unbiased)	7.99482
Variance (unbiased)	8.27633
Standard Deviation (unbiased)	2.87686
Coefficient of Variation (unbiased)	3.50914
Squared Differences between all Pairs of Observations	16.5527
Mean Absolute Differences between all Pairs of Observations	1.41523
Gini Mean Difference	1.41523
Leik Measure of Dispersion	0.59021
Index of Diversity	0.881053
Index of Qualitative Variation	0.889063
Coefficient of Dispersion	n/a

n/a not available.

**Table 17:** Variability analysis of the Australia data.

Absolute range	22
Relative range (unbiased)	9.82118
Variance (unbiased)	5.01785
Standard Deviation (unbiased)	2.24006
Coefficient of Variation (unbiased)	2.28116
Squared Differences between all Pairs of Observations	10.0357
Mean Absolute Differences between all Pairs of Observations	1.33202
Gini Mean Difference	1.33202
Leik Measure of Dispersion	0.53995
Index of Diversity	0.944533
Index of Qualitative Variation	0.95312
Coefficient of Dispersion	0.920055

**Table 18:** Variability analysis of the Africa data.

Absolute range	5
Relative range (unbiased)	5.3562
Variance (unbiased)	0.871417
Standard Deviation (unbiased)	0.933497
Coefficient of Variation (unbiased)	2.52727
Squared Differences between all Pairs of Observations	1.74283
Mean Absolute Differences between all Pairs of Observations	0.649304
Gini Mean Difference	0.649304
Leik Measure of Dispersion	0.512639
Index of Diversity	0.933968
Index of Qualitative Variation	0.942458
Coefficient of Dispersion	n/a

n/a not available.

## References

[1] Burgess T.F., Shaw N.E. (2010). Editorial board membership of management and business journals: a social network analysis study of the Financial Times 40. Br. J. Manag..

[2] Nisonger T. (2002). The relationship between international editorial board composition and citation measures in political science, business, and genetics journals. Scientometrics.

[3] Ò. Miró, P. Burbano, C.A. Graham, D.C. Cone, J. Ducharme, A.F.T. Brown, F.J. Martín-Sánchez, Analysis of h-index and other Bibliometric Markers of Productivity and Repercussion of a Selected Sample of Worldwide Emergency Medicine Researchers. 10.1136/emermed-2016-205893 27565195

[4] Vošner H.B., Kokol P., Bobek S., Železnik D., Završnik J. (2016). A bibliometric retrospective of the journal computers in human behavior (1991–2015). Comput. Hum. Behav..

[5] Petersen J., Hattke F., Vogel R. (2017). Editorial governance and journal impact: a study of management and business journals. Scientometrics.

[6] Garg K.C., Pali S. (2016). A preliminary investigation of editorial gatekeeping of CSIR-NISCAIR journals. Ann. Libr. Info. Stud..

[7] Jokić M., Sirotić G. (2016). Do the international editorial board members of croatian social sciences and humanities journals contribute to their visibility?. Medij-. Istraz..

[8] Wicherts J.M. (2016). Peer review quality and transparency of the peer-review process in open access and subscription journals. PLoS One.

[9] Metz I., Harzing A.W., Zyphur M.J. (2016). Of journal editors and editorial boards: who are the trailblazers in increasing editorial board gender equality?. Br. J. Manag..

[10] Cummings S., Hoebink P. (2017). Representation of academics from developing countries as authors and editorial board members in scientific journals: does this matter to the field of development studies?. Eur. J. Dev. Res..

[11] Rösing C.K., Junges R., Haas A.N. (2014). Publication rates of editorial board members in oral health journals. Braz. Oral Res..

[12] Schisterman E.F., Swanson C.W., Lu Y.L., Mumford S.L. (2017). The changing face of epidemiology: gender disparities in citations?. Epidemiology.

[13] Dhanani A., Jones M.J., M. J (2017). Editorial boards of accounting journals: gender diversity and internationalisation. Account. Audit. Account. J..

[14] Petersen J. (2017). How innovative are editors?: evidence across journals and disciplines. Res. Eval..

[15] Shideler G.S., Araújo R.J. (2017). Reviewer interest in a manuscript may predict its future citation potential. Scientometrics.

[16] Sarigöl E.E., Garcia D., Scholtes I., Schweitzer F. (2017). Quantifying the effect of editor–author relations on manuscript handling times. Scientometrics.

[17] O.T. Kayode, H.I. Okagbue, J.A. Achuka, Water Quality Assessment for Groundwater Around a Municipal Waste Dumpsite, Data in Brief, 〈10.1016/j.dib.2018.01.072〉. 10.1016/j.dib.2018.01.072 PMC5852294 29552607

[18] Okagbue H.I., Opanuga A.A., Oguntude P.E., Ugwoke P.O. (2017). Random number datasets generated from statistical analysis of randomly sampled GSM recharge cards. Data Brief.

[19] Wessa P. (2016). Variability (v1.0.7) in Free Statistics Software (v1.2.1). http://www.wessa.net/rwasp_variability.wasp/.

[20] Okagbue H.I., Adamu M.O., Oguntunde P.E., Opanuga A.A., Owoloko E.A., Bishop S.A. (2017). Datasets on the statistical and algebraic properties of primitive Pythagorean triples. Data Brief.

[21] J.Y. Park, Z. Nagy, Bibliography data for thermal comfort and building control research – Keywords co-occurrences relationship and citation network from 5536 articles, Data in Brief, 〈https://doi.org/10.1016/j.dib.2018.01.033〉.

